# Antibiotic Resistance Modulation and Modes of Action of (-)-α-Pinene in *Campylobacter jejuni*

**DOI:** 10.1371/journal.pone.0122871

**Published:** 2015-04-01

**Authors:** Jasna Kovač, Katarina Šimunović, Zuowei Wu, Anja Klančnik, Franz Bucar, Qijing Zhang, Sonja Smole Možina

**Affiliations:** 1 Department of Food Science and Technology, Biotechnical Faculty, University of Ljubljana, Ljubljana, Slovenia; 2 Department of Veterinary Microbiology and Preventive Medicine, College of Veterinary Medicine, Iowa State University, Ames, United States of America; 3 Department of Pharmacognosy, Institute of Pharmaceutical Sciences, University of Graz, Graz, Austria; Institut National de la Recherche Agronomique, FRANCE

## Abstract

The aim of the study was to investigate the mode of action of (-)-α-pinene in terms of its modulation of antibiotic resistance in *Campylobacter jejuni*. Broth microdilution and ethidium bromide accumulation assays were used to evaluate the (-)-α-pinene antimicrobial activity, modulation of antimicrobial resistance, and inhibition of antimicrobial efflux. The target antimicrobial efflux systems were identified using an insertion mutagenesis approach, and *C*. *jejuni* adaptation to (-)-α-pinene was evaluated using DNA microarrays. Knock-out mutants of the key up-regulated transcriptional regulators *hspR* and *hrcA* were constructed to investigate their roles in *C*. *jejuni* adaptation to several stress factors, including osmolytes, and pH, using Biolog phenotypical microarrays. Our data demonstrate that (-)-α-pinene efficiently modulates antibiotic resistance in *C*. *jejuni* by decreasing the minimum inhibitory concentrations of ciprofloxacin, erythromycin and triclosan by up to 512-fold. Furthermore, (-)-α-pinene promotes increased expression of *cmeABC* and another putative antimicrobial efflux gene, *Cj1687*. The ethidium bromide accumulation was greater in the wild-type strain than in the antimicrobial efflux mutant strains, which indicates that these antimicrobial efflux systems are a target of action of (-)-α-pinene. Additionally, (-)-α-pinene decreases membrane integrity, which suggests that enhanced microbial influx is a secondary mode of action of (-)-α-pinene. Transcriptomic analysis indicated that (-)-α-pinene disrupts multiple metabolic pathways, and particularly those involved in heat-shock responses. Thus, (-)-α-pinene has significant activity in the modulation of antibiotic resistance in *C*. *jejuni*, which appears to be mediated by multiple mechanisms that include inhibition of microbial efflux, decreased membrane integrity, and metabolic disruption. These data warrant further studies on (-)-α-pinene to develop its use in the control of antibiotic resistance in *Campylobacter*.

## Introduction

The increasing incidence of bacterial pathogens that show antibiotic resistance has led to impaired efficacy of clinical treatments, prolonged illness and greater mortality. This rising antimicrobial resistance has been accompanied by a decline in new antibiotic discovery over the last few decades, which now poses a serious threat to public health. In Europe it has been estimated that 25,000 patients die annually due to incurable infections with multidrug-resistant (MDR) bacteria, and that the healthcare system is burdened with at least an additional 1.5 billion euros each year because of the prolonged treatments that are needed for infections with MDR bacteria [1]. *Campylobacter jejuni* is the most prevalent bacterial cause of gastroenteritis, and it shows high rates of ciprofloxacin resistance; as such, *C*. *jejuni* represents an important part of this healthcare burden [2, 3]. Drug resistant *Campylobacter* are on the CDC list of serious threats in the U.S., which further indicates the importance of *Campylobacter* in public health [4]. *Campylobacter* resistance to quinolone antibiotics is especially problematic, because quinolone resistance has increased globally and tends to spread clonally [5].

One of the major mechanisms that contributes to the resistance of MDR bacteria is enhanced antimicrobial efflux, which extrudes antimicrobials of out of bacterial cells with broad specificity. The most important antimicrobial efflux pump in *C*. *jejuni* is CmeABC, while CmeDEF and CmeG have secondary roles [6–8]. Alone or in combination with specific point mutations and antibiotic resistance genes, these antimicrobial efflux pumps provide increased resistance to clinically important classes of antibiotics.

To restore the activity of antibiotics that are already available on the market, research has been devoted to finding new compounds with activities that can be used to inhibit antimicrobial efflux in bacteria [9–12]. However, to date, no inhibitors of these antimicrobial efflux pumps have been licensed for clinical use, although some drugs, such as the calcium ion influx inhibitor verapamil that is licensed for arrhythmia treatment, also show inhibitory activity against antimicrobial efflux. Also, some new natural products have recently been identified for potential use as antimicrobial efflux pump inhibitors, based on findings from *in-vitro* studies [13–17].

One of these natural compounds that has been shown to have antimicrobial activity against various microorganisms is the monoterpene α-pinene, which is naturally present in various essential oils [18]. α-Pinene is also one of the constituents of an essential oil from *Alpinia katsumadai* seeds (J. Kovač, unpublished data), which we have shown to have modulatory activity towards antimicrobial resistance in *C*. *jejuni* and *Staphylococcus aureus* [10, 13]. α-Pinene exists naturally as both (+)-α-pinene and (-)-α-pinene [11]. (-)-α-Pinene was investigated here, in terms of its activity as an antimicrobial, its modulation of antimicrobial resistance, and its inhibition of antimicrobial efflux, using antibiotic-susceptible and antibiotic-resistant *C*. *jejuni* isolates from different sources. Furthermore, the *C*. *jejuni* responses to treatment with (-)-α-pinene were studied using transcriptomic and phenotypic microarray approaches.

## Materials and Methods

### Chemicals

Erythromycin, ciprofloxacin, ethidium bromide (EtBr), carbonyl cyanide-m-chlorophenylhydrazone (CCCP), reserpine, (-)-α-pinene, resazurin sodium salt, and menadion were from Sigma-Aldrich Chemie (Steinheim, Germany), triclosan and chloramphenicol were from Calbiochem (Merck KGaA, Darmstadt, Germany), ampicillin was from Roche Diagnostics (Mannheim, Germany), and kanamycin was from Merck (Darmstadt, Germany).

### Bacterial strains and growth conditions

Frozen stocks (from -80°C storage) of the *C*. *jejuni* strains listed in S1 Table were cultured on selective Karmali agar and Mueller-Hinton agar (Oxoid, Hampshire, UK), or in Mueller-Hinton broth (Oxoid), and incubated at 42°C under microaerobic conditions (5% O_2_, 10% CO_2_, in N_2_). *Escherichia coli* were cultured on Luria-Bertani agar (Oxoid) at 37°C. When needed, the Mueller-Hinton agar was supplemented with kanamycin (30 mg/L) or chloramphenicol (4 mg/L), and the Luria-Bertani agar was supplemented with ampicillin (50 mg/L).

### Antimicrobial and resistance-modulation assays

The antimicrobial activity of (-)-α-pinene was determined on 17 *C*. *jejuni* strains and two *C*. *jejuni* mutants with knocked-out antimicrobial efflux genes (Δ*cmeB* and Δ*Cj1687*), using the broth microdilution method. Briefly, the tested compounds were dissolved in dimethylsulphoxide (final concentration, 2.5%) and serially diluted in Mueller-Hinton broth in black microtitre plates, and the *C*. *jejuni* cultures were added at a concentration of 5 ×10^5^ CFU/mL, to the final volume of 0.1 mL/well. After 24 h incubation at 42°C under microaerobic conditions, 10 μL resazurin reagent was added to each well, which consisted of 10 mM tetrazolium salts and 0.8 mM menadion. Following a 2-h incubation at 42°C, the fluorescence intensity was measured at 550 nm and 959 nm, using a microplate reader (Tecan, Mannedorf/Zurich, Switzerland) [19]. The minimum inhibitory concentrations (MICs) were defined as the minimal concentrations at which the fluorescence signal declined to the level of the blank. Modulation of antimicrobial resistance was evaluated using the same method, for nine *C*. *jejuni* strains and two knocked-out antimicrobial efflux mutants (Δ*cmeB* and Δ*Cj1687*), except that the medium was supplemented with subinhibitory concentrations of (-)-α-pinene (62.5 mg/L or 125 mg/L) for the determination of the MICs of ciprofloxacin, erythromycin, triclosan, and ethidium bromide. The (-)-α-pinene modulation factors were defined as the ratios of the MICs for the antimicrobial alone and for the antimicrobial in the presence of (-)-α-pinene. A modulation factor >2 was set as the cut-off for biologically significant resistance modulation [14].

### Membrane integrity

The influence of 62.5 mg/L and 125 mg/L (-)-α-pinene on membrane integrity of *C*. *jejuni* NCTC 11168 was followed using LIVE/DEAD BacLight Bacterial Viability kits (L-7012; Molecular Probes, Eugene, Oregon, USA) [20]. A mixture of the green fluorescent dye SYTO 9 and propidium iodide was prepared and used according to the manufacturer instructions (Molecular Probes). This dye mixture was added to 100 μL *C*. *jejuni* cultures (OD_600_, 0.2; 1:1, v/v) that were untreated or treated with (-)-α-pinene. The kinetics of propidium iodide intracellular penetration were followed by measuring the relative fluorescence units (RFUs) in 60-s intervals over 1 h, in terms of the SYTO 9 fluorescence at 481 nm and 510 nm in a microplate reader (Tecan). Two independent experiments were carried out in triplicate in black microtitre plates. The membrane disruption (%) was calculated from the kinetics measurements of the treated relative to the untreated cultures over the last 10 min of the assay.

### Ethidium bromide accumulation

The influence of 62.5 mg/L and 125 mg/L (-)-α-pinene on EtBr accumulation in *C*. *jejuni* NCTC 11168, Δ*cmeB* and Δ*Cj1*6*87* was determined. Further screening was carried out with 62.5 mg/L (-)-α-pinene on 16 *C*. *jejuni* isolates (S1 Table), as previously described [13]. Briefly, exponential phase cultures were washed and resuspended in phosphate-buffered saline (OD_600_, 0.2). After a 10-min incubation at 37°C, the efflux pump inhibitors were added to 100 μL cultures in black microtitre plates, and incubated for 10 min at 37°C before adding EtBr to a final the concentration of 0.5 mg/L. The kinetics of intracellular EtBr accumulation were measured at 500 nm and 608 nm using a Tecan microplate reader, at 45-s intervals for 1 h. In parallel to the (-)-α-pinene treatments, the reference efflux pump inhibitors CCCP (10 mg/L) and reserpine (100 mg/L) were tested. The selected concentrations of the efflux pump inhibitors had no inhibitory effects on bacterial growth. Other EPIs, such as verapamil, NMP, and PaβN were also tested in the preliminary experiments, but they were excluded from further studies due to their low activity (data not shown). Measurements were carried out in triplicate and the means of the last 10 measurements were used in the statistical analysis.

### Transcriptional response to (-)-α-pinene

The influence of (-)-α-pinene on gene expression in *C*. *jejuni* NCTC 11168 was determined using DNA microarrays and qRT-PCR. Exponential phase cultures were adjusted to OD_600_ 0.2 in Mueller-Hinton broth. Five millilitres of culture were treated with 62.5 mg/L (-)-α-pinene dissolved in dimethylsulphoxide. Only dimethylsulphoxide (0.048%) was added to the untreated samples. The cultures were incubated for 2 h, microaerobically and with shaking (160 rpm), at 42°C. Experiments were carried out as four biological replicates. RNA Protect Bacteria reagent (Qiagen, Maryland, USA) was added to the cultures, and the total RNA was isolated using RNeasy mini kits (Qiagen), and treated with Ambion Turbo DNA-*free* kits (Invitrogen, USA).

Microarrays with 4,751 probes that targeted 1,756 genes that are specific to *C*. *jejuni* NCTC 11168 (Mycroarray, Biodiscovery-LLC, MI, USA) were used for the gene-expression analysis, as described previously [21]. The cDNA was synthesised with random hexamers, SuperScript III Reverse Transcriptase, and aminoallyl dUTP (all supplied by Invitrogen, USA) and labelled with a monofunctional NHS-ester dye, as Cy3 or Cy5 (GE Healthcare, Buckinghamshire, UK). The DNA concentration and the labelling efficiency were determined spectrophotometrically (NanoDrop 1000; Thermo Scientific, Waltham, MA, USA). Four biological replicates were hybridised to four microarrays, according to the manufacturer protocol, and incubated for 24 h at 42°C, and scanned at 550 nm for Cy3 and 650 nm for Cy 5, using a microarray scanner (GenePix 4100A; Molecular Devices, Sunnyvale, CA, USA), to 5 μm resolution. The fluorescence intensities were converted to digital signals using the GenepixPro 7.0 programme (MolecularDevices, Sunnyvale, CA, USA). The microarray data was deposited with the NCBI Gene Expression Omnibus, under the accession number GSE59879, GPL19011.

The expression of four genes was confirmed by quantitative real-time PCR using a real-time PCR detection system (ABI 7500; Applied Biosystems, Thermo Scientific, Waltham, MA, USA) with Universal KAPA SYBR One-Step qRT-PCR kits (Kapa Biosystems, Boston, USA) and the same RNA template as in the microarray experiments. Quantitative RT-PCR primers (Supplementary Information S2 Table) were designed using the online tool Primer3 (http://frodo.wi.mit.edu/). Standard quantification curves were generated for each of the RNA templates, using 10-fold dilution series between 25 ng/μL and 0.0025 ng/μL. Quantitation was performed in 15 μL reactions with three technical replicates of three biological replicates, according to the following programme: 10 min at 50°C, 5 min at 60°C, 3 min at 95°C and 40 cycles of 10 s at 95°C and 30 s at 58°C, including the melt curve analysis performed after amplification. Here, 16S rRNA was used for the internal normalisation standard for the calculation of the relative fold-change in gene expression, by the comparative threshold cycle method (ΔΔCt) [22].

### Construction of insertional mutants

**Fig 1:**
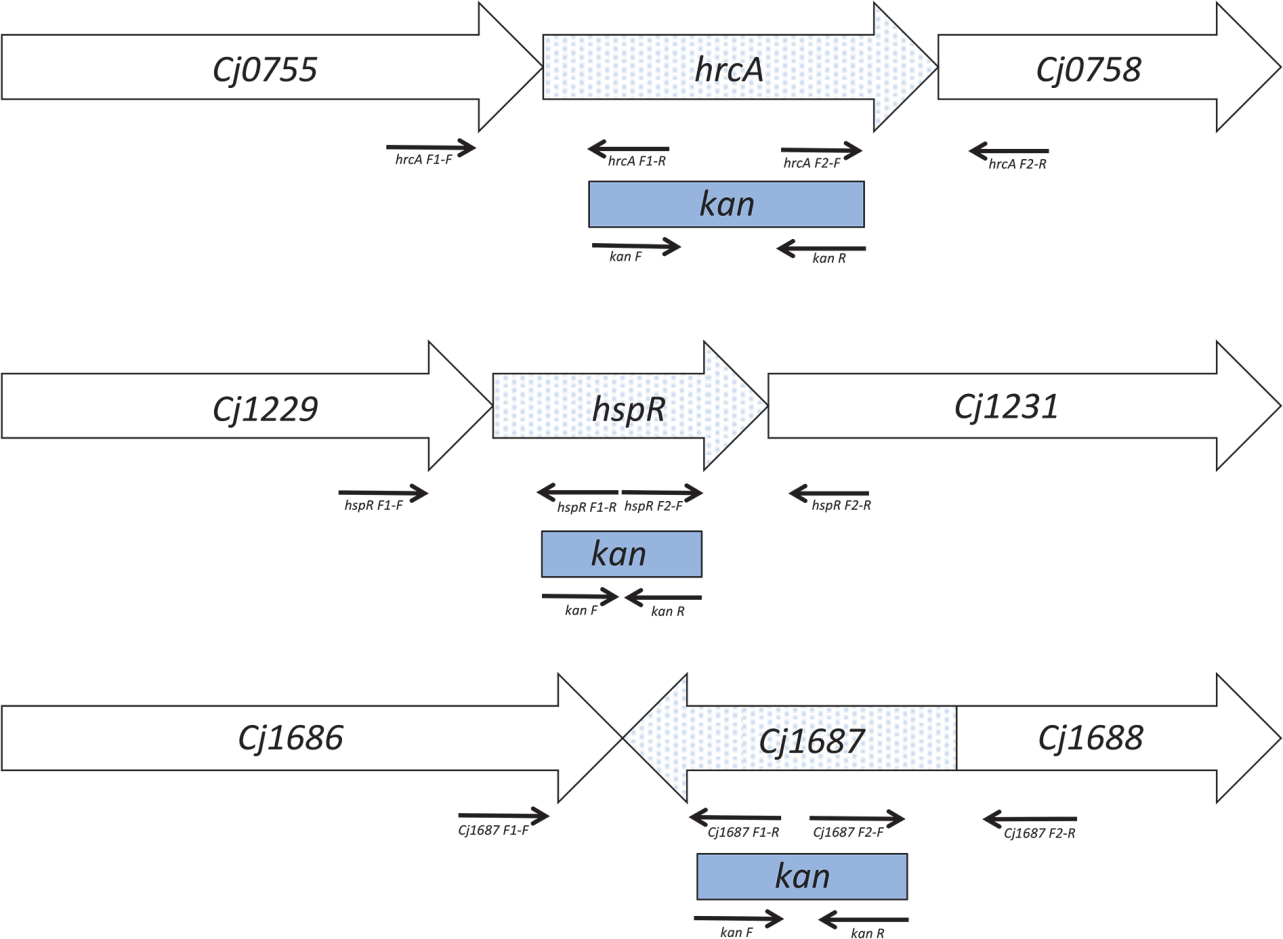
Scheme of the insertional mutagenesis strategies (not to scale). Box arrows, target open reading frames; solid arrows, primers used for DNA fragment amplification (from left to right: F1-F, F1-R, F2-F, F2-R, specific for each individual mutant, as listed in S2 Table); blue dots, target gene; blue rectangles, deleted regions substituted with a kanamycin cassette (*kan*).

Three *C*. *jejuni* NCTC 11168 knock-out mutants with disrupted open reading frames of the *hspR*, *hrcA* and *Cj1687* genes were constructed (S2 Table) using an insertion mutagenesis approach. The internal fragments of the genes were replaced with the kanamycin resistance cassette *aphA-3* (Fig 1). The PCR products used for construction of these mutants were amplified with GoTaq polymerase (Promega Corporation, Madison, WI, USA) using the primers listed in S2 Table. Following amplification, these were purified using a Wizard SV gel and PCR clean-up system (Promega), and digested with HindIII or PstI (Thermo Scientific, Waltham, MA, USA), or both. The restricted PCR products were incubated at 80°C, to deactivate the restriction enzymes, ligated using Quick ligation kits (New England Biolabs, Ipswich, MA, USA), and cloned into pGEM-T Easy Vectors using T4 DNA ligase (Promega). The plasmid was amplified in competent *E*. *coli* DH5α, isolated using GenElute Plasmid Miniprep kits (Sigma, St. Luis, MO, USA), and transformed into *C*. *jejuni* NCTC 11168 by natural transformation. The transformants with the insert were selected on Mueller-Hinton agar supplemented with kanamycin (30 mg/L), and confirmed with PCR (S2 Table).

### Metabolic response to inactivated genes

*C*. *jejuni* NCTC 11168 and the Δ*hspR* and Δ*hrcA* mutants were prepared in IF-0a inoculating fluid (Biolog, Hayward, CA, USA), mixed with redox indicator Dye D, according to the manufacturer instructions, and dispensed (100 μL/well) using carbon utilisation, osmolality, and pH phenotypic plates PM1, PM2A, PM9 and PM10 (Biolog). The PM plates were placed in plastic bags together with microaerophilic atmosphere generating sachets (Oxoid), sealed and fixated in the automatic plate reader (Omnilog; Biolog) trays using adhesive tape. Plates were incubated in automatic plate reader at 42°C and the metabolism of different carbon sources, growth on different osmolytes and at different pHs was followed spectrophotometrically every 15 min for 48 h. The endpoint values were used in the comparative statistical analysis.

### Statistical analysis

The statistical significances of the data for the antimicrobial actions, modulation of antimicrobial resistance, and EtBr accumulation were determined using one-way ANOVA with appropriate *post-hoc* tests, using the SPSS software, version 21 **(IBM Corp., Armonk, NY, USA)**. Analysis of the Biolog phenotypic microarray data was carried out using MS Excel and Student’s t-tests, and analysis of the DNA microarray data was performed with the R software using the LIMMA package, as previously described [21, 23]. Statistical significance was set at p <0.05, and the cut-off for significance of the relative fold-changes in the modulation of antimicrobial resistance set at >2, the gene expression between untreated and treated cultures set at ⩾2, and in the growth on phenotypic plates set at ⩾1.5.

## Results

### Antimicrobial activity and modulation of antimicrobial resistance of (-)-α-pinene

The antimicrobial activity of (-)-α-pinene was determined on 16 strains of *C*. *jejuni* (S1 Table). The MICs of (-)-α-pinene were 1000 mg/L or higher in all of the tested strains, including those tested in the antimicrobial-resistance-modulation assay (Table 1). These high MICs confirmed the insignificant antimicrobial activity of (-)-α-pinene. As a potential modulator of antimicrobial resistance, (-)-α-pinene was tested at 62.5 mg/L and 125 mg/L in combination with the antibiotics ciprofloxacin and erythromycin, the disinfectant triclosan, and the antimicrobial efflux pump substrate EtBr, on the *C*. *jejuni* NCTC 11168 reference strain. At 62.5 mg/L, (-)-α-pinene decreased the MICs of these antimicrobials by up to 2-fold. However, at 125 mg/L, (-)-α-pinene reduced the MICs of the various antimicrobials from 32-fold to over 512-fold (Table 1).

**Table 1:** Antimicrobial activity and modulation of antimicrobial resistance of (-)-α-pinene for the different strains of *Campylobacter jejuni*.

***C*. *jejuni***	MIC (mg/L)												
strain	AP	CIP			ERY			TC			EtBr		
		-AP	+AP	MF	-AP	+AP	MF	-AP	+AP	MF	-AP	+AP	MF
NCTC 11168*	1000	0.063	0.031	2	0.5	0.25	2	8	4	2	1	1	1
NCTC 11168	1000	0.063	<0.002	>32	0.5	<0.002	>256	8	<0.125	>64	1	<0.008	>128
K49/4	2000	0.063	0.002	32	0.25	<0.002	>128	4	0.25	16	2	0.016	128
53124	1000	4	2	2	0.25	0,063	4	4	4	1	0.5	0.5	1
375/06	2000	4	1	4	0.125	<0.008	>16	4	<0.125	>32	0.25	<0.008	>32
57360	1000	4	<0.008	>512	0.25	<0.002	>128	8	<0.063	>128	0.25	<0.008	>32
58429	1000	4	<0.031	>128	0.25	<0.125	>2	4	<0.125	>32	0.25	<0.063	>4
60089	>2000	8	2	4	0.125	0.063	2	32	32	1	8	8	1
1518/08	2000	8	<0.031	>256	0.125	<0.002	>64	16	<0.031	>512	0.5	<0.008	>64
573/03	2000	16	4	4	0.125	0.063	2	16	<0.063	>256	0.5	<0.008	>64
ΔcmeB	1000	0.016	<0.001	>16	0.063	<0.002	>32	8	<0.063	>128	0.063	<0.008	>8
Δ*Cj1687*	2000	0.063	<0.001	>63	0.125	<0.002	>64	8	<0.063	>128	0.5	<0.008	>64

MIC, minimal inhibitory concentration; AP, (-)-α-pinene; CIP, ciprofloxacin; ERY: erythromycin; TC, triclosan; EtBr, ethidium bromide;-AP, in the absence of (-)-α-pinene; +AP, with 125 mg/L (-)-α-pinene, MF, modulation factor.

* In this case +AP was applied in concentration 62.5 mg/L.

Due to the significantly stronger modulation of antimicrobial resistance of (-)-α-pinene at 125 mg/L, this higher concentration was further tested on eight other *C*. *jejuni* isolates and two mutants with inactivated antimicrobial efflux genes, Δ*cmeB* and Δ*Cj1687* (Table 1). These data indicated pronounced modulation of antimicrobial resistance by (-)-α-pinene with all of these strains for most of the antimicrobials tested. Significant MIC reductions were produced also in the Δ*cmeB* and Δ*Cj1687* mutants derived from the wild type NCTC 11168, which suggest that this modulatory activity of 125 mg/L (-)-α-pinene is not dependent on its inhibition of antimicrobial efflux.

### Influence of (-)-α-pinene on membrane integrity

**Fig 2:**
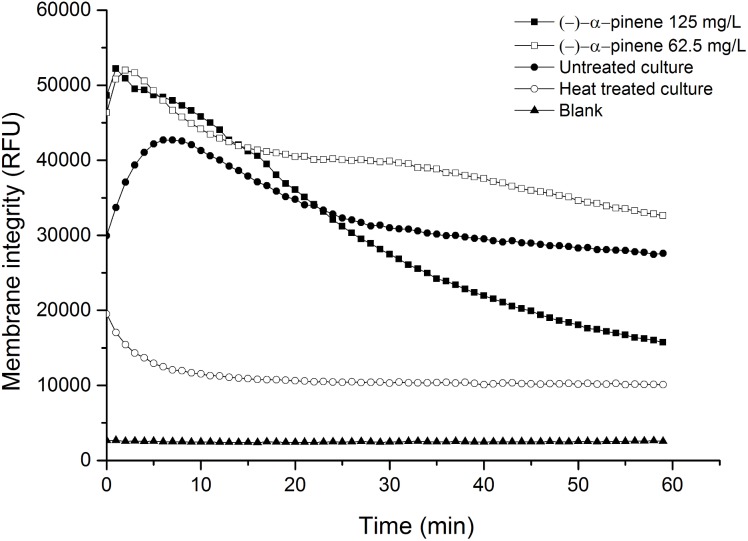
Influence of (-)-α-pinene on membrane integrity of *C*. *jejuni* NCTC 11168. Membrane integrity of wild-type *C*. *jejuni* NCTC 11168 cultures, untreated culture, culture treated with 125 mg/L (-)-α-pinene or 62.5 mg/L (-)-α-pinene, heat treated culture and blank.

As for the modulation of antimicrobial resistance, both 62.5 mg/L and 125 mg/L (-)-α-pinene were tested for their influence on membrane integrity in *C*. *jejuni*, to determine whether membrane permeability is the main mechanism of its modulation of antimicrobial resistance. The membrane integrity of cultures treated with 62.5 mg/L (-)-α-pinene was 20% higher than that of the untreated cultures after 1-h treatment, while at 125 mg/L (-)-α-pinene, the membrane integrity was decreased by 39% (Fig 2). Hence, at this higher concentration of 125 mg/L (-)-α-pinene, the consequent disruption of the membranes is likely to have contributed to its modulation of antimicrobial resistance. Cultures incubated at 80°C for 15 min were used as a positive control for disrupted membranes, and these showed 64% decreased membrane integrity, compared to the untreated control cultures. These differences were calculated based on the kinetics measurements over the last 10 min of the 1-h assays, and they were statistically significant (p <0.0001). The disruptive impact of (-)-α-pinene on membrane integrity is not surprising, as it is known that monoterpenes can cause alterations in membrane permeability [24].

### Inhibition of antimicrobial efflux by (-)-α-pinene

**Fig 3:**
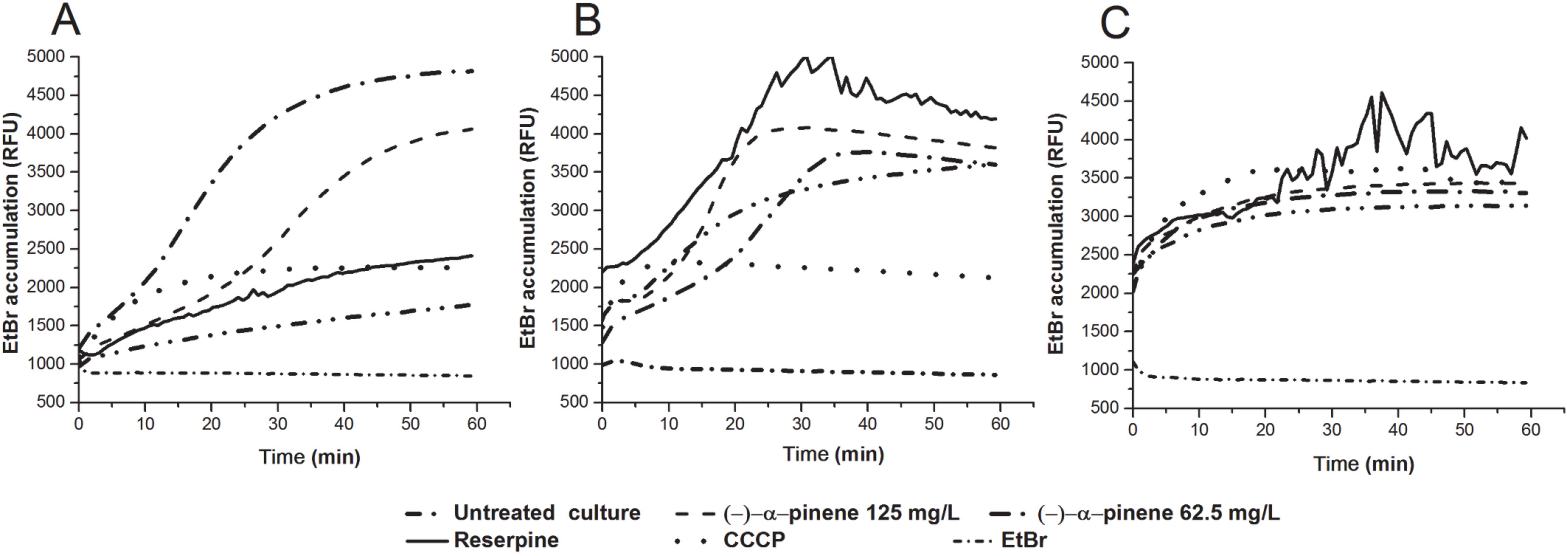
Influence of (-)-α-pinene, CCCP and reserpine on ethidium bromide accumulation. (A) Reference strain *C*. *jejuni* NCTC 11168, (B) *C*. *jejuni* NCTC 11168 ΔcmeB knock-out mutant, (C) *C*. *jejuni* NCTC 11168 Δ*Cj1687* knock-out mutant.

**Fig 4:**
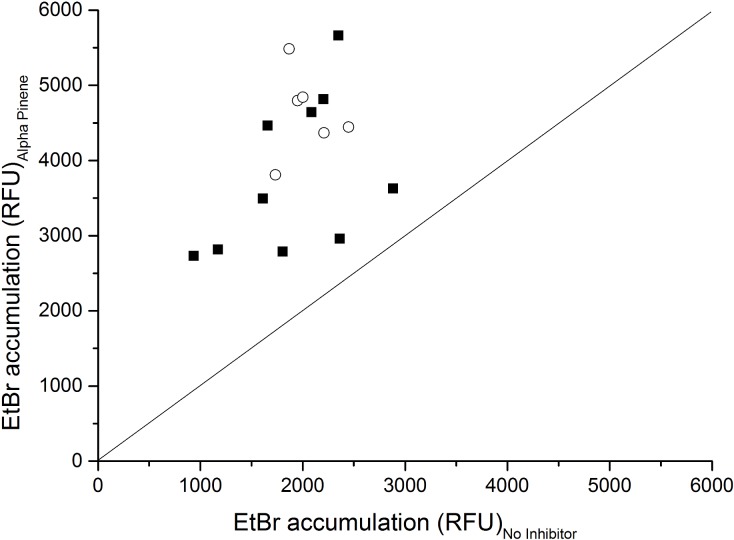
Influence of (-)-α-pinene on EtBr accumulation in the 16 *C*. *jejuni* strains. Relationship between EtBr accumulation in untreated cultures and in cultures treated with 62.5 mg/L (-)-α-pinene. Each symbol represents a single isolate. Solid squares, antibiotic resistant strains (resistant to three or more unrelated antibiotics); open circles, antibiotic sensitive strains.

Again, both 62.5 mg/L and 125 mg/L (-)-α-pinene were tested for their impact on intracellular EtBr accumulation in *C*. *jejuni* NCTC 11168 and the two mutants (Δ*cmeB* and Δ*Cj1687*). Compared to the non-treated control, both of these concentrations of (-)-α-pinene increased the EtBr accumulation; however, 62.5 mg/L (-)-α-pinene resulted in greater EtBr accumulation (Fig 3). As 62.5 mg/L (-)-α-pinene did not have a negative impact on membrane integrity, this was used in the further screening on a larger set of 16 isolates (S1 Table). This enabled the investigation of only the inhibitory effects of (-)-α-pinene on antimicrobial efflux. Here, there was increased EtBr accumulation in all of these 16 tested strains when 62.5 mg/L (-)-α-pinene was added (Fig 4). This (-)-α-pinene concentration increased the EtBr accumulation significantly more than seen for CCCP and reserpine in all of these tested strains, which confirms its inhibitory activity against antimicrobial efflux. Here it is important to note that (-)-α-pinene was tested at a lower concentration (62.5 mg/L) than reserpine (100 mg/L), and at a higher concentration than CCCP (10 mg/L), to avoid the toxic effects of CCCP.

To identify the target efflux system of (-)-α-pinene, EtBr accumulation in the two mutants in the antimicrobial efflux genes was investigated. In comparison with the control wild-type *C*. *jejuni* NCTC 11168, the increase in EtBr accumulation with 62.5 mg/L (-)-α-pinene was significantly lower in the Δ*cmeB* and Δ*Cj1687* mutants (Fig 3), although this remained above the EtBr accumulation in the wild-type. This finding suggests that (-)-α-pinene inhibits both of these antimicrobial efflux systems.

### Transcriptional response to (-)-α-pinene

After a 2-h treatment with 62.5 mg/L (-)-α-pinene, 128 genes of *C*. *jejuni* NCTC 11168 were differentially expressed (with the cut-off set at ≥2-fold difference; Table 2 and S3 Table). Of those, 108 were up-regulated, and 20 were down-regulated. The Clusters of Orthologous Groups functional classification of these differentially expressed genes revealed that the up-regulated genes were mainly involved in amino-acid transport and metabolism (12 genes), translation (12 genes), inorganic ion transport and metabolism (8 genes), posttranslational modification and chaperones (8 genes), and unknown functions (36 genes, which included ‘poorly characterised’, ‘function unknown’, ‘general function prediction only’). The down-regulated genes were scattered into 12 of the functional categories (Table 2). Interestingly, however, the most strongly up-regulated genes (>10-fold) fell into the groups of posttranslational modification and chaperones, and transcription, which included the chaperone-encoding genes *grpE*, *dnaK*, and *clpB*, and the heat-shock regulator *hrcA*. In these two functional categories, another two heat-shock-response genes that encode the chaperones *groES* and *cbpA* and the transcriptional repressor *hspR* were also up-regulated, although to lower levels (2-fold to 4-fold). As well as these heat-shock-response genes, the antimicrobial efflux pump genes *cmeABC* were up-regulated by 2.7-fold to 2.2-fold, respectively. Another, as yet uncharacterised gene that encodes a putative antimicrobial efflux protein, *Cj1687*, was 2.5-fold up-regulated after the treatment with (-)-α-pinene. The *omp50* gene that was previously identified as an outer membrane protein and a phosphotyrosine kinase [25] was 4.9-fold up-regulated here. Several ribosomal genes (*rps*, *rpl*) and genes involved in purine biosynthesis (*purM*, *apt*, *purD*), glutamate metabolism (*murI*, *metE*, *metF*, *glnA*, *gltBD*), and iron–sulphur homeostasis (*chuAD*, *tonB3*, *tonB2*) were expressed at higher levels (2.1-fold to 8.1-fold up-regulated) after the treatment with (-)-α-pinene. On the other hand, aspartate-ammonia lyase (*aspA*) was the most strongly down-regulated (4.6-fold) metabolic gene, together with the genes that encode succinate dehydrogenase (*sdhA*, *sdhC*), fumarate hydratase (*fumC*), and anaerobic C4-dicarboxylate antiporter *dcuA*, which were up to 3.2-fold down-regulated following the treatment with (-)-α-pinene.

**Table 2:** Functional categories of the differentially expressed genes in *Campylobacter jejuni*.

Clusters of orthologous groups category	Up-regulated genes	Down-regulated gens	Differentially expressed genes
Energy production and conversion	2	3	5
Amino acid transport and metabolism	12	1	13
Nucleotide transport and metabolism	5	0	5
Carbohydrate transport and metabolism	1	2	3
Coenzyme transport and metabolism	4	1	5
Lipid transport and metabolism	2	0	2
Translation	12	0	12
Transcription	3	2	5
Replication, recombination and repair	5	0	5
Cell wall/ membrane biogenesis	5	2	7
Cell motility	1	0	1
Posttranslational modification, chaperones	8	3	11
Inorganic ion transport and metabolism	8	1	9
General function prediction only	9	2	11
Function unknown	1	1	2
Signal transduction mechanisms	1	1	2
Defense mechanisms	3	1	4
Poorly characterized	26	0	26
**Total**	**108**	**20**	**128**

### Metabolic response to inactivated genes

The *hspR*, *hrcA* mutants and wild-type *C*. *jejuni* NCTC 11168 were grown on Biolog phenotype MicroArrays with different carbon sources and in presence of different osmolytes and pH, to investigate their roles in adaptation to these stresses. The growth of the mutants was compared to the growth of the wild-type strain (S4 Table). The *hspR* mutant grew better on some of the osmolytes, such as 6% NaCl with KCl, sodium sulphate, sodium formate, sodium lactate, and sodium phosphate, while growth of the *hrcA* mutant, on the other hand, was inhibited by these and several of the other osmolytes. The *hrcA* mutant did not use a wide range of the carbon sources as efficiently as the wild-type.

## Discussion

(-)-α-pinene has negligible anti-*Campylobacter* activity, with MICs of 1000 mg/L or higher against all of the tested strains. Unlike (+)-α-pinene, this (-)-α-pinene enantiomer was also previously reported to have no inhibitory effects in fungi and with *S*. *aureus* [11]. Although (-)-α-pinene did not inactivate *C*. *jejuni* growth, it showed remarkable modulatory activity of antimicrobial resistance when applied in combination with ciprofloxacin, erythromycin, triclosan and ethidium bromide. We observed concentration-dependent activity of (-)-α-pinene here, and investigated the mechanisms of its action at 62.5 mg/L and 125 mg/L. Although the modulation of antimicrobial resistance was substantially more pronounced at 125 mg/L (-)-α-pinene, the EtBr accumulation remained at similarly high levels.

These data suggest the involvement of at least two different modes of action. Indeed, investigation of the membrane integrity revealed increased membrane permeability with 125 mg/L (-)-α-pinene. At this concentration of (-)-α-pinene, the synergistic effects of increased membrane permeability and intracellular accumulation due to the inhibition of antimicrobial efflux provided the best modulation of antimicrobial resistance.

Previous reports have demonstrated the modulation of antimicrobial resistance and efflux of the diarylheptanoid and flavonoid constituents of *A*. *katsumadai* seeds in *Mycobacterium smegmatis* at comparable concentrations, although its terpene compounds were studied only as a mixture, with the essential oil at significantly higher concentrations [13, 14]. Inhibition of antimicrobial efflux was also previously achieved in *M*. *smegmatis* and *S*. *aureus*, with paradol-related and gingerol-related compounds isolated from *Aframomum melegueta*, and with a novel antibacterial natural product that was isolated from *Hypericum olympicum* L. cf. *uniflorum*, respectively [15, 17].

To determine the antimicrobial efflux system that (-)-α-pinene targets, we carried out comparative accumulation assays in the wild-type and with the mutant strains with inactivated antimicrobial efflux genes, Δ*cmeB* and Δ*cj1687*. In the presence of (-)-α-pinene, the decrease in EtBr accumulation almost to the level of the untreated culture in Δ*cmeB* and Δ*Cj1687* suggests that these two efflux proteins are the likely the targets of (-)-α-pinene.

To date, (-)-α-pinene is the first plant-derived compound that shown inhibition of antimicrobial efflux in *Campylobacter* to be described and characterized. Cytotoxicity of α-pinene was previously investigated with the HeLa and Cos7 cell lines, where IC_50_ values of 357.9 mg/L and 337.5 mg/L were reported, respectively [26]. As these IC_50_ values for α-pinene are more than 2-fold greater than those used in the present modulation assays, and more than 5-fold greater than those used in the present EtBr accumulation assays, (-)-α-pinene has the potential to be further investigated as a means for the control of antibiotic resistant *C*. *jejuni*.

To explore the *C*. *jejuni* responses to (-)-α-pinene, we analysed the changes in the *C*. *jejuni* transcriptome before and after the 2-h (-)-α-pinene treatment. Here, we observed increases in the expression of the *cmeABC* and *Cj1687* antimicrobial efflux genes that were identified as potential targets for antimicrobial efflux inhibition, which confirms the increased need for CmeABC and Cj1687 activity after inhibition by (-)-α-pinene. The involvement of (-)-α-pinene in alterations to membrane permeability, as a complementary mechanism of its action, was also indicated by the increased expression of the outer membrane protein product of the *omp50* gene. This protein was previously shown to modulate phosphorylation of proteins involved in capsule production [25]. Up-regulation of *omp50* appears to be a strategy for bacterial adaptation, as *opm50* is likely to enhance the production of surface polysaccharides, and thereby to contribute to decreased membrane permeability and decreased antimicrobial influx [25].

Similar responses of *C*. *jejuni* were also observed previously after exposure to erythromycin, which like (-)-α-pinene, is a hydrophobic compound [27]. Furthermore, treatment of *C*. *jejuni* with (-)-α-pinene evoked increased expression of ribosomal and purine biosynthesis genes, which indicated that there were metabolic changes, and thereby the associated need for increased protein synthesis. Previous reports have indicated similar transcriptional changes in *C*. *jejuni* in response to stomach acid, which evoked up-regulation of *purB* [28]. The product of the up-regulated glutamate synthetase gene (*gltBD*) catalyses the condensation of glutamate, which is involved in osmoadaptation and can be further converted to aspartate. Then, as well as coding for heat-shock proteins, up-regulation of the *gltD* and *glnA* genes was previously reported in *C*. *jejuni* in response to salt stress [29]. Aspartate-ammonia lyase (*aspA*), which is the most strongly down-regulated gene associated with energy metabolism, cleaves carbon–nitrogen bonds and catalyses the conversion of L-aspartate into fumarate and ammonia. Additionally, down-regulation of succinate dehydrogenase genes (*sdhA*, *sdh*) contributes to inhibition of fumarate biosynthesis, obstruction of oxidative energy metabolism, and conversion of fumarate to malate by fumarate hydratase (*fumC*). Metabolic changes and an imbalance in Krebs cycle intermediates were also reflected in the down-regulation of *fumC* and the anaerobic C4-dicarboxylate antiporter gene *dcuA*. Up-regulation of iron–sulphur homeostasis genes, such as *chuAD*, *tonB3* and *tonB2*, suggested an increase in the transport of the metal ions and siderophores that are involved in respiration and DNA synthesis.

The changes in gene expression caused by (-)-α-pinene are strikingly similar to those that have previously been observed in a mutant with a defective transcriptional regulator *hspR*, which showed highly up-regulated heat-shock-related genes: *grpE*, *dnaK*, *clpB* and *hrcA* [30]. Similar responses were also observed after treatment with the natural antimicrobial compound benzyl isothiocyanate, which promotes protein aggregation [31]. The simple heat-shock-response model from a study by Holmes et al. (2010) suggested repression of *groES* and *groEL* by *hrcA*, and repression of *dnaK*, *cbpA* and the *clpB* operon by *hspR*, which become derepressed in the event of heat shock, allowing the expression of *hrcA* [30]. HrcA, however, needs the assistance of *groES* and *groEL* for posttranscriptional modifications that enable its activity [30].

Characterisation of mutant strains using phenotypic microarrays revealed increased adaptation of Δ*hspR* to increased concentrations of several sodium salts. On the other hand, the growth of the mutant Δ*hrcA* was attenuated in the presence of a wide variety of osmolytes and high pH. The Δ*hrcA* mutant also did not use a wide range of carbon sources, compared to the wild-type. These data demonstrate that the heat-shock transcriptional regulators are also important for efficient responses to stresses other than high temperatures.

In conclusion, (-)-α-pinene is here confirmed as an efficient modulator of antimicrobial resistance in *C*. *jejuni*, with at least two different mechanisms that contribute synergistically to this activity. Lower concentrations of (-)-α-pinene show pronounced inhibition of antimicrobial efflux through the targeting of the main efflux system CmeABC and another, as yet uncharacterised, efflux protein, Cj1687. The higher concentration of (-)-α-pinene additionally targeted the membrane, with increased permeability, thereby promoting the influx of antimicrobials. The low antimicrobial activity that we observed appears to be derived from the effective bacterial adaptation to (-)-α-pinene treatment provided by the heat-shock response, and efficient changes in protein synthesis and energy metabolism. Due to the promising modulation of antimicrobial resistance and its previously reported low cytotoxicity, (-)-α-pinene has the potential to be further investigated for the control of antimicrobial resistant in *C*. *jejuni*.

## Supporting Information

S1 Table Bacterial strains and plasmids used in the study. (DOCX)

S2 Table Primers used for mutant construction and qRT-PCR. (DOCX)

S3 Table Differentially expressed genes in (-)-α-pinene treated *C*. *jejuni* NCTC 11168. (DOCX)

S4 Table Influence of deactivation of *hspR* and *hrcA* on *Campylobacter jejuni* use of different carbon sources (Biolog PM1 and PM2) and growth in presence of different osmolytes and pH (Biolog PM9 and PM10). (DOCX)

## References

[1] European Center for Disease Prevention and Control EMA. The bacterial challenge: time to react ECDC/EMEA 2009 Available: http://www.ecdc.europa.eu/en/publications/Publications/0909_TER_The_Bacterial_Challenge_Time_to_React.pdf.

[2] EFSA. The European Union summary report on trends and sources of zoonoses, zoonotic agents and food-borne outbreaks in 2012. EFSA Journal 2014; 12(2):3547. 10.2903/j.efsa.2018.5500 PMC7009540 32625785

[3] EFSA. The European Union summary report on antimicrobial resistance in zoonotic and indicator bacteria from humans, animals and food in 2011. EFSA Journal 2014;12(3):3590. 10.2903/j.efsa.2020.6007 PMC7448042 32874244

[4] Prevention CDC (2013) Antibiotic resistance threats in the United States CDC: 1–114. Available: http://www.cdc.gov/drugresistance/threat-report-2013/pdf/ar-threats-2013-508.pdf.

[5] Kovač J, Čadez N, Lušicky M, Nielsen EM, Ocepek M, Raspor P, et al The evidence for clonal spreading of quinolone resistance with a particular clonal complex of *Campylobacter jejuni* . Epidemiology and Infection 2014;13: 1–9. 10.1017/S0950268813003245 PMC9151304 24534165

[6] Jeon B, Wang Y, Hao H, Barton YW, Zhang Q. Contribution of CmeG to antibiotic and oxidative stress resistance in *Campylobacter jejuni* . The Journal of Antimicrobial Chemotherapy 2011; 66: 79–85. 10.1093/jac/dkq418 21081547 PMC3001851

[7] Akiba M, Lin J, Barton YW, Zhang Q. Interaction of CmeABC and CmeDEF in conferring antimicrobial resistance and maintaining cell viability in *Campylobacter jejuni* . The Journal of Antimicrobial Chemotherapy 2006; 57: 52–60. 16303882 10.1093/jac/dki419

[8] Lin J, Michel LO, Zhang Q. CmeABC functions as a multidrug efflux system in *Campylobacter jejuni* . Antimicrobial Agents and Chemotherapy 2002; 46: 2124–2131. 12069964 10.1128/AAC.46.7.2124-2131.2002 PMC127319

[9] Klančnik A, Možina SS, Zhang Q. Anti-Campylobacter activities and resistance mechanisms of natural phenolic compounds in Campylobacter PLoS One. 2012; 7: e51800 10.1371/journal.pone.0051800 23284770 PMC3524091

[10] Klančnik A, Groblacher B, Kovač J, Bucar F, Možina SS. Anti-*Campylobacter* and resistance-modifying activity of *Alpinia katsumadai* seed extracts. Journal of Applied Microbiology 2012; 113: 1249–1262. 10.1111/j.1365-2672.2012.05424.x 22897164

[11] da Silva AC, Lopes PM, de Azevedo MM, Costa DC, Alviano CS, Alviano DS. Biological activities of alpha-pinene and beta-pinene enantiomers. Molecules 2012; 17: 6305–6316. 10.3390/molecules17066305 22634841 PMC6268778

[12] Wang W, Li N, Luo M, Zu Y, Efferth T. Antibacterial activity and anticancer activity of Rosmarinus officinalis L. essential oil compared to that of its main components. Molecules 2012; 17: 2704–2713. 10.3390/molecules17032704 22391603 PMC6268287

[13] Kovač J GN, Bucar F, Smole Možina S. Antimicrobial and resistance modulatory activity of *Alpinia katsumadai* seed extract, essential oil and post-distillation extract. Food Technology and Biotechnology 2014; 52: 248–254.

[14] Groblacher B, Kunert O, Bucar F. Compounds of *Alpinia katsumadai* as potential efflux inhibitors in *Mycobacterium smegmatis* . Bioorganic and Medicinal Chemistry 2012; 20: 2701–2706. 10.1016/j.bmc.2012.02.039 22459211

[15] Shiu WK, Malkinson JP, Rahman MM, Curry J, Stapleton P, Gunaratnam M et al A new plant-derived antibacterial is an inhibitor of efflux pumps in *Staphylococcus aureus* . International Journal of Antimicrobial Agents 2013; 42: 513–518. 10.1016/j.ijantimicag.2013.08.007 24119569

[16] Kurinčič M, Klančnik A, Smole Možina S. Epigallocatechin gallate as a modulator of *Campylobacter* resistance to macrolide antibiotics. International Journal of Antimicrobial Agents 2012; 40: 467–471. 10.1016/j.ijantimicag.2012.07.015 22999765

[17] Groblacher B, Maier V, Kunert O, Bucar F. Putative mycobacterial efflux inhibitors from the seeds of *Aframomum melegueta* . Journal of Natural Products 2012; 75: 1393–1399. 10.1021/np300375t 22789014

[18] Teixeira B MA, Ramos C, Neng NR, Nogueira JMF, Saraiva JA, Nunes ML. Chemical composition and antibacterial and antioxidant properties of commercial essential oils. Industrial Crops and Products 2013; 43: 587–595.

[19] Tsukatani T, Suenaga H, Higuchi T, Akao T, Ishiyama M, Ezoe K et al Colorimetric cell proliferation assay for microorganisms in microtiter plate using water-soluble tetrazolium salts. Journal of Microbiological Methods 2008; 75: 109–116. 10.1016/j.mimet.2008.05.016 18586343

[20] Simoes M, Rocha S, Coimbra MA, Vieira MJ. Enhancement of *Escherichia coli* and *Staphylococcus aureus* antibiotic susceptibility using sesquiterpenoids. Medicinal Chemistry 2008; 4: 616–623. 18991747 10.2174/157340608786242016

[21] Wu Z, Sahin O, Shen Z, Liu P, Miller WG, Zhang Q. Multi-omics approaches to deciphering a hypervirulent strain of *Campylobacter jejuni* . Genome Biology and Evolution 2013; 5: 2217–2230. 10.1093/gbe/evt172 24201373 PMC3845652

[22] Livak KJ, Schmittgen TD. Analysis of relative gene expression data using real-time quantitative PCR and the 2(-Delta Delta C(T)) Method. Methods 2001; 25: 402–408. 11846609 10.1006/meth.2001.1262

[23] Smyth GK. Linear models and empirical bayes methods for assessing differential expression in microarray experiments. Statistical Applications in Genetics and Molecular Biology. 2004; 3: Article3. 10.2202/1544-6115.1027 16646809

[24] Trombetta D, Castelli F, Sarpietro MG, Venuti V, Cristani M, Daniele C et al Mechanisms of antibacterial action of three monoterpenes. Antimicrobial Agents and Chemotherapy 2005; 49: 2474–2478. 15917549 10.1128/AAC.49.6.2474-2478.2005 PMC1140516

[25] Corcionivoschi N, Alvarez LA, Sharp TH, Strengert M, Alemka A, Mantell J et al Mucosal reactive oxygen species decrease virulence by disrupting *Campylobacter jejuni* phosphotyrosine signaling. Cell Host & Microbe 2012; 12: 47–59. 22817987 10.1016/j.chom.2012.05.018 PMC3749511

[26] Herrmann F, Wink M. Synergistic interactions of saponins and monoterpenes in HeLa cells, Cos7 cells and in erythrocytes. Phytomedicine 2011; 18: 1191–1196. 10.1016/j.phymed.2011.08.070 21968386

[27] Xia Q, Muraoka WT, Shen Z, Sahin O, Wang H, Wu Z et al Adaptive mechanisms of *Campylobacter jejuni* to erythromycin treatment. BMC Microbiology 2013; 13: 133 10.1186/1471-2180-13-133 23767761 PMC3694039

[28] Reid AN, Pandey R, Palyada K, Whitworth L, Doukhanine E, Stintzi A et al Identification of *Campylobacter jejuni* genes contributing to acid adaptation by transcriptional profiling and genome-wide mutagenesis. Applied and Environmental Microbiology 2008; 74: 1598–1612. 10.1128/AEM.01508-07 18192408 PMC2258640

[29] Cameron A, Frirdich E, Huynh S, Parker CT, Gaynor EC. Hyperosmotic stress response of *Campylobacter jejuni* . Journal of Bacteriology. 2012; 194: 6116–6130. 10.1128/JB.01409-12 22961853 PMC3486418

[30] Holmes CW, Penn CW, Lund PA. The *hrcA* and *hspR* regulons of *Campylobacter jejuni* . Microbiology 2010; 156: 158–166. 10.1099/mic.0.031708-0 19850618

[31] Dufour V, Stahl M, Rosenfeld E, Stintzi A, Baysse C. Insights into the mode of action of benzyl isothiocyanate on *Campylobacter jejuni* . Applied and Environmental Microbiology 2013; 79: 6958–6968. 10.1128/AEM.01967-13 24014524 PMC3811535

