# Supplementary material for: Antibiotic Resistance Modulation and Modes of Action of (-)-α-Pinene in Campylobacter jejuni
Source: PLoS One. 2015 Apr 1;10(4):e0122871. doi: 10.1371/journal.pone.0122871 (PMC4382180; doi:10.1371/journal.pone.0122871)
Supplement: S1 Table — (DOCX) [file pone.0122871.s001.docx]

**S1 Table**. Bacterial strains and plasmids used in the study.

| **Bacterial strain/ plasmid** | **Designation** | **Description** | **Reference** |
| --- | --- | --- | --- |
| Bacterial strains | NCTC 11168 | *Campylobacter jejuni*, human isolate | NCTC |
|  | ATCC 33560 | *Campylobacter jejuni*, animal isolate | ATCC |
|  | K49/4 | *Campylobacter jejuni*, meat isolate | 5 |
|  | 58429 | *Campylobacter jejuni*, meat isolate | 5 |
|  | 53124 | *Campylobacter jejuni,* meat isolate | 5 |
|  | 57360 | *Campylobacter jejuni*, meat isolate | 5 |
|  | 60089 | *Campylobacter jejuni,* meat isolate | 5 |
|  | 9581 | *Campylobacter jejuni*, human isolate | 5 |
|  | 9090 | *Campylobacter jejuni,* human isolate | 5 |
|  | 9711 | *Campylobacter jejuni*, human isolate | 5 |
|  | 1190/09 | *Campylobacter jejuni,* animal isolate | 5 |
|  | 375/06 | *Campylobacter jejuni*, human isolate | 5 |
|  | 573/03 | *Campylobacter jejuni,* meat isolate | 5 |
|  | 1518/08 | *Campylobacter jejuni*, animal isolate | 5 |
|  | C2 | *Campylobacter jejuni,* meat isolate | 5 |
|  | C33 | *Campylobacter jejuni*, meat isolate | 5 |
|  | 816 | *Campylobacter jejuni,* water isolate | 5 |
|  | 660/08 | *Campylobacter jejuni*, animal isolate | 5 |
|  | 11168 Δ*cmeB* | *NCTC 11168* Δ*cmeB* knock-out mutant | 9 |
|  | 11168 Δ*Cj1687* | *NCTC 11168* Δ*Cj1687* knock-out mutant | This study |
|  | 11168 Δ*hspR* | *NCTC 11168* Δ*hspR* knock-out mutant | This study |
|  | 11168 Δ*hrcA* | *NCTC 11168* Δ*hrcA* knock-out mutant | This study |
|  | DH5α | *Escherichia coli*, competent strain | Promega |
| Plasmids | pGEM^®^-T Easy Vector | Cloning vector, ampiciline resistance | Promega |
|  | pGEMΔ*Cj1687* | Δ*Cj1687* cloned into pGEM®-T Easy Vector | This study |
|  | pGEMΔ*hspR* | Δ*hspR*cloned into pGEM®-T Easy Vector | This study |
|  | pGEMΔ*hrcA* | Δ*hrcA* cloned into pGEM®-T Easy Vector | This study |
