# Supplementary material for: Antibiotic Resistance Modulation and Modes of Action of (-)-α-Pinene in Campylobacter jejuni
Source: PLoS One. 2015 Apr 1;10(4):e0122871. doi: 10.1371/journal.pone.0122871 (PMC4382180; doi:10.1371/journal.pone.0122871)
Supplement: S2 Table — (DOCX) [file pone.0122871.s002.docx]

**S2 Table.** Primers used for mutant construction and qRT-PCR.

| **Purpose** | **Primer code** | **Sequence (5' → 3')** | **Reference** |
| --- | --- | --- | --- |
| Species | CJF | ACTTCTTTATTGCTTGCTGC | 9 |
| confirmation | CJR | GCCACAACAAGTAAAGAAGC | 9 |
|  | CCF | GTAAAACCAAAGCTTATCGTG | 9 |
|  | CCR | TCCAGCAATGTGTGCAATG | 9 |
|  | CLF | TAGAGAGATAGCAAAAGAGA | 9 |
|  | CLR | TACACATAATAATCCCACCC | 9 |
|  | CUF | AATTGAAACTCTTGCTATCC | 9 |
|  | CUR | TCATACATTTTACCCGAGCT | 9 |
|  | CFF | GCAAATATAAATGTAAGCGGAGAG | 9 |
|  | CFR | TGCAGCGGCCCCACCTAT | 9 |
|  | 23SF | TATACCGGTAAGGAGTGCTGGAG | 9 |
|  | 23SR | ATCAATTAACCTTCGAGCACCG | 9 |
| Mutant | *hrcA* F1-F | ATGCAAGTGGGAGTGGTAGC | This study |
| construction | *hrcA* F1-R | CGCGGATCCCGAAGCAGGTATGCAAAGGT | This study |
|  | *hrcA* F2-F | GAACTGCAGTATGGGGCTTAAGGTGGATG | This study |
|  | *hrcA* F2-R | GCACTTGAACCACTTCACCA | This study |
|  | *hspR* F1-F | TGGAGGATTTGGAGGTTTTG | This study |
|  | *hspR* F1-R | CGCGGATCCTTGCCTTAAGGTTTGTGGATG | This study |
|  | *hspR* F2-F | GAACTGCAGCGGCTAGTAAAGCCGTTGTT | This study |
|  | *hspR* F2-R | AAAAGCAAAATTCCCAATGC | This study |
|  | *Cj1687* F1-F | TCTTTGGCATCTTTGGCTTT | This study |
|  | *Cj1687* F1-R | CGCGGATCCTAGCAGGCAGAGCAGATGAA | This study |
|  | *Cj1687* F2-F | GAACTGCAGAATCGCCTTAGCTTTGCTTG | This study |
|  | *Cj1687* F2-R | TTATCCCTGGAATTCGTCCA | This study |
|  | *kan* F | CGCGGATCCCGCTTATCAATATATCTATAGAATGG | This study |
|  | *kan* R | GAACTGCAGGATAATGCTAAGACAATCACTAAAG | This study |
| qRT-PCR | *dnaK-*F | TGGCGCATCAAGTAGAAAAA | This study |
|  | *dnaK-*R | TCACGCAAATCATCAAGAGC | This study |
|  | *grpE-*F | TGAAAAACATGGAGTGGCTCT | This study |
|  | *grpE-*R | GCACTTGAACCACTTCACCA | This study |
|  | *aspA*-F | TGGGGAATTGGAAATCTCTG | This study |
|  | *aspA*-R | CCCTAACAAAGCGAGGAAAA | This study |
|  | *dcuA-*F | CAACAAGCAGGTGGACTTGA | This study |
|  | *dcuA-*R | TTGTAAGCAACCACCCACAA | This study |
