# Supplementary material for: Antibiotic Resistance Modulation and Modes of Action of (-)-α-Pinene in Campylobacter jejuni
Source: PLoS One. 2015 Apr 1;10(4):e0122871. doi: 10.1371/journal.pone.0122871 (PMC4382180; doi:10.1371/journal.pone.0122871)
Supplement: S3 Table — (DOCX) [file pone.0122871.s003.docx]

**S3 Table.** Differentially expressed genes in (-)-α-pinene treated *C. jejuni* NCTC 11168.

| **Gene ID** | | **Functional category** | **Fold change** | |
| --- | --- | --- | --- | --- |
|  | |  | **Microarray** | **qRT-PCR** |
| *Cj0757* | *hrcA* | Putative heat shock regulator | 25.8 |  |
| *Cj0509c* | *clpB* | ATP-dependent Clp protease ATP-binding subunit | 18.3 |  |
| *Cj0759* | *dnaK* | Heat-shock protein | 14.7 | 6.11 |
| *Cj0758* | *grpE* | Heat-shock protein | 11.9 | 6.95 |
| *Cj1373* |  | Putative ntegral membrane protein | 8.2 |  |
| *Cj0699c* | *glnA* | Glutamine synthetase | 8.1 |  |
| *Cj0567* |  | Hypothetical protein | 8 |  |
| *Cj0908* |  | Putative periplasmic protein | 7.8 |  |
| *Cj0970* |  | Hypothetical protein | 7 |  |
| *Cj0561c* |  | Putative periplasmic protein | 6.8 |  |
| *Cj1622* | *ribD* | Riboflavin-specific deaminase/reductase | 6.3 |  |
| *Cj0790* | *purU* | Formyltetrahydrofolate deformylase | 6 |  |
| *Cj0030* |  | Hypothetical protein | 5.9 |  |
| *Cj0037c* |  | Putative cytochrome C | 5.5 |  |
| *Cj0013* | *ilvD* | Dihydroxy-acid dehydratase | 5.3 |  |
| *Cj0121* |  | Conserved hypothetical protein | 5.3 |  |
| *Cj1410c* |  | Putative membrane protein | 5.2 |  |
| *Cj0022c* |  | Putative ribosomal pseudouridine synthase | 5.1 |  |
| *Cj0007* | *gltB* | Glutamate synthase (NADPH) large subunit | 5 |  |
| *Cj0945c* |  | Putative helicase | 4.9 |  |
| *Cj1170c* | *omp50* | 50-kDa outer membrane protein precursor | 4.9 |  |
| *Cj0321* | *dxs* | L-deoxy-D-xylulose-5-phosphate synthase | 4.8 |  |
| *Cj1001* | *rpoD* | RNA polymerase sigma factor (sigma-70) | 4.8 |  |
| *Cj1229* | *cbpA* | Putative curved-DNA binding protein | 4.7 |  |
| *Cj1652c* | *murI* | Glutamate racemase | 4.7 |  |
| *Cj1103* | *csrA* | Carbon storage regulator homolog | 4.5 |  |
| *Cj1230* | *hspR* | Heat-shock transcriptional regulator | 4.1 |  |
| *Cj1169c* |  | Putative periplasmic protein | 3.9 |  |
| *Cj0816* |  | Hypothetical protein | 3.7 |  |
| *Cj1250* | *purD* | Phosphoribosylamine-glycine ligase | 3.7 |  |
| *Cj0692c* |  | Putative membrane protein | 3.4 |  |
| *Cj1713* |  | Putative radical SAM domain protein | 3.4 |  |
| *Cj0456c* |  | Hypothetical protein | 3.2 |  |
| *Cj0163c* |  | Hypothetical protein | 3 |  |
| *Cj0262c* |  | Putative methyl-accepting chemotaxis signal | 3 |  |
| *Cj0849c* |  | Conserved hypothetical protein | 3 |  |
| *Cj1303* | *fabH2* | Putative 3-oxoacyl-[acyl-carrier-protein] | 3 |  |
| *Cj1584c* |  | Putative peptide ABC-transport system | 3 |  |
| *Cj0198c* |  | Helicase-like protein | 2.9 |  |
| *Cj0733* |  | Putative HAD-superfamily hydrolase | 2.9 |  |
| *Cj1101* |  | ATP-dependent DNA helicase | 2.9 |  |
| *Cj1201* | *metE* | 5-Methyltetrahydropteroyltriglutamate-- | 2.9 |  |
| *Cj0008* |  | Conserved hypothetical protein | 2.8 |  |
| *Cj0009* | *gltD* | Glutamate synthase (NADPH) small subunit | 2.8 |  |
| *Cj0293* | *surE* | Multifunctional protein SurE homolog | 2.8 |  |
| *Cj0485* |  | Putative oxidoreductase | 2.8 |  |
| *Cj0957c* |  | Hypothetical protein | 2.8 |  |
| *Cj0348* | *trpB* | Tryptophan synthase beta chain | 2.7 |  |
| *Cj0367c* | *cmeA* | Periplasmic fusion protein CmeA | 2.7 |  |
| *Cj0772c* |  | Putative NLPA family lipoprotein | 2.7 |  |
| *Cj1082c* | *thiD* | Phosphomethylpyrimidine kinase | 2.7 |  |
| *Cj1553c* | *hsdM* | Putative type I restriction enzyme M protein | 2.7 |  |
| *Cj1614* | *chuA* | Haemin uptake system outer membrane receptor | 2.7 |  |
| *Cj0927* | *apt* | Adenine phosphoribosyltransferase | 2.6 |  |
| *Cj1199* |  | Putative iron/ascorbate-dependent | 2.6 |  |
| *Cj1242* |  | Hypothetical protein | 2.6 |  |
| *Cj0366c* | *cmeB* | Inner membrane efflux transporter CmeB | 2.5 |  |
| *Cj0494* |  | Putative exporting protein | 2.5 |  |
| *Cj1472c* |  | Putative membrane protein | 2.5 |  |
| *Cj1630* | *tonB2* | Putative TonB transport protein | 2.5 |  |
| *Cj1687* |  | Putative efflux protein | 2.5 |  |
| *Cj0017c* | *dsbI* | Disulphide bond formation protein | 2.4 |  |
| *Cj0018c* | *dba* | Disulphide bond formation protein | 2.4 |  |
| *Cj0391c* |  | Hypothetical protein | 2.4 |  |
| *Cj0706* |  | Conserved hypothetical protein | 2.4 |  |
| *Cj0989* |  | Putative membrane protein | 2.4 |  |
| *Cj1202* | *metF* | 5.10-Methylenetetrahydrofolate reductase | 2.4 |  |
| *Cj1231* | *kefB* | Putative glutathione-regulated potassium-efflux | 2.4 |  |
| *Cj1255* |  | Putative isomerase | 2.4 |  |
| *Cj1479c* | *rpsI* | 30S ribosomal protein S9 | 2.4 |  |
| *Cj1529c* | *purM* | Phosphoribosylformylglycinamidine cyclo-ligase | 2.4 |  |
| *Cj0419* |  | Putative histidine triad (HIT) family protein | 2.3 |  |
| *Cj0667* |  | Putative S4 domain protein | 2.3 |  |
| *Cj0727* |  | Putative periplasmic solute-binding protein | 2.3 |  |
| *Cj0859c* |  | Hypothetical protein | 2.3 |  |
| *Cj0963* |  | Hypothetical protein | 2.3 |  |
| *Cj1200* |  | Putative NLPA family lipoprotein | 2.3 |  |
| *Cj1298* |  | Putative N-acetyltransferase | 2.3 |  |
| *Cj1388* |  | Putative endoribonuclease L-PSP | 2.3 |  |
| *Cj1480c* | *rplM* | 50S ribosomal protein L13 | 2.3 |  |
| *Cj1659* | *p19* | Periplasmic protein p19 | 2.3 |  |
| *Cj1718c* | *leuB* | 3-Isopropylmalate dehydrogenase | 2.3 |  |
| *Cj0145* |  | Putative TAT (Twin-Arginine Translocation) | 2.2 |  |
| *Cj0365c* | *cmeC* | Outer membrane channel protein CmeC | 2.2 |  |
| *Cj0415* |  | Putative GMC oxidoreductase subunit | 2.2 |  |
| *Cj0453* | *thiC* | Thiamin biosynthesis protein | 2.2 |  |
| *Cj0753c* | *tonB3* | TonB transport protein | 2.2 |  |
| *Cj1583c* |  | Putative peptide ABC-transport system permease | 2.2 |  |
| *Cj1707c* | *rplC* | 50S ribosomal protein L3 | 2.2 |  |
| *Cj1708c* | *rpsJ* | 30S ribosomal protein S10 | 2.2 |  |
| *Cj1717c* | *leuC* | 3-Isopropylmalate dehydratase large subunit | 2.2 |  |
| *Cj0080* |  | Putative membrane protein. Functional | 2.1 |  |
| *Cj0414* |  | Putative oxidoreductase subunit | 2.1 |  |
| *Cj0518* | *htpG* | Hsp90 family heat shock protein | 2.1 |  |
| *Cj0568* |  | Hypothetical protein | 2.1 |  |
| *Cj0736* |  | Hypothetical protein | 2.1 |  |
| *Cj1070* | *rpsF* | 30S ribosomal protein S6 | 2.1 |  |
| *Cj1072* | *rpsR* | 30S ribosomal protein S18 | 2.1 |  |
| *Cj1181c* | *tsf* | Elongation factor TS | 2.1 |  |
| *Cj1617* | *chuD* | Putative haemin uptake system periplasmic | 2.1 |  |
| *Cj1704c* | *rplB* | 50S ribosomal protein L2 | 2.1 |  |
| *Cj1706c* | *rplD* | 50S ribosomal protein L4 | 2.1 |  |
| *Cj0146c* | *trxB* | Thioredoxin reductase | 2 |  |
| *Cj0295* |  | Putative acetyltransferase | 2 |  |
| *Cj1071* | *ssb* | Single-strand DNA binding protein | 2 |  |
| *Cj1220* | *groES* | 10 kDa chaperonin (cpn10) | 2 |  |
| *Cj1658* |  | Putative iron permease | 2 |  |
| *Cj1721c* |  | Putative outer membrane protein | 2 |  |
| *Cj0360* | *glmM* | Phosphoglucosamine mutase | -2 |  |
| *Cj0069* |  | Hypothetical protein | -2 |  |
| *Cj0361* | *lspA* | Lipoprotein signal peptidase | -2.1 |  |
| *Cj0362* |  | Putative integral membrane protein | -2.1 |  |
| *Cj1226c* |  | Putative two-component sensor (histidine | -2.1 |  |
| *Cj1227c* |  | Putative two-component regulator | -2.1 |  |
| *Cj1279c* |  | Putative fibronectin domain-containing | -2.1 |  |
| *Cj1364c* | *fumC* | Fumarate hydratase | -2.1 |  |
| *Cj1131c* | *gne* | UDP-GlcNAc/Glc 4-epimerase | -2.2 |  |
| *Cj0440c* |  | Putative transcriptional regulator | -2.3 |  |
| *Cj0596* | *peb4-cbf2* | Major antigenic peptide PEB-cell binding factor | -2.4 |  |
| *Cj0597* | *fba* | Fructose-bisphosphate aldolase | -2.4 |  |
| *Cj0950c* |  | Putative lipoprotein | -2.4 |  |
| *Cj1380* |  | Putative periplasmic protein | -2.5 |  |
| *Cj1130c* | *pglK* | Flippase | -2.6 |  |
| *Cj0437* | *sdhA* | Succinate dehydrogenase flavoprotein subunit | -3.2 |  |
| *Cj0439* | *sdhC* | Putative succinate dehydrogenase subunit C | -3.3 |  |
| *Cj0088* | *dcuA* | Anaerobic C4-dicarboxylate transporter | -3.6 | -7.5 |
| *Cj0358* |  | Putative cytochrome C551 peroxidase | -3.6 |  |
| *Cj0087* | *aspA* | Aspartate ammonia-lyase | -4.6 | -7.1 |
