# Supplementary material for: Antibiotic Resistance Modulation and Modes of Action of (-)-α-Pinene in Campylobacter jejuni
Source: PLoS One. 2015 Apr 1;10(4):e0122871. doi: 10.1371/journal.pone.0122871 (PMC4382180; doi:10.1371/journal.pone.0122871)
Supplement: S4 Table — (DOCX) [file pone.0122871.s004.docx]

**S4 Table.** Influence of deactivation of *hspR* and *hrcA* on *Campylobacter jejuni* use of different carbon sources (Biolog PM1 and PM2) and growth in presence of different osmolytes and pH (Biolog PM9 and PM10).

| **Growth on substrate relative to wild type CJ NCTC 11168** | | | |
| --- | --- | --- | --- |
|  | **CJ NCTC 111168Δ*hspR*** |  | **CJ NCTC 111168Δ*hrcA*** |
| ↑ | Glycerol | ↑ | L-Mallic acid |
| ↑ | Pyruvic acid | ↑ | 3-0-β-D-Galactopyranosyl-D-arabinose |
| ↑ | 1% NaCl | ↓ | L-arabinose |
| ↑ | 6.5% NaCl | ↓ | Succinic acid |
| ↑ | 6% NaCl + creatinine | ↓ | D-Galactose |
| ↑ | 6% NaCl + KCl | ↓ | L-Aspartic acid |
| ↑ | 6% NaCl + glycerol | ↓ | L-Proline |
| ↑ | 6% NaCl + octopine | ↓ | D-Mannose |
| ↑ | 4% Potassium chloride | ↓ | D-Serine |
| ↑ | 2% Sodium sulphate | ↓ | L-Fucose |
| ↑ | 3% Sodium sulphate | ↓ | D-Glucoronic acid |
| ↑ | 1% Sodium formate | ↓ | D-Xylose |
| ↑ | 4% Sodium formate | ↓ | Formic acid |
| ↑ | 5% Sodium formate | ↓ | L-Glutamic acid |
| ↑ | 1% Sodium lactate | ↓ | D-Glucose-6-phosphate |
| ↑ | 9% Sodium lactate | ↓ | D-Galactonic acid-γ-lactone |
| ↑ | 100 mM Sodium phosphate pH 7 | ↓ | D-Robose |
| ↓ | Glycolic acid | ↓ | L-Rhamnose |
| ↓ | Glycyl-L-proline | ↓ | D-Fructose |
| ↓ | Tyramine | ↓ | Acetic acid |
| ↓ | D-Lactic acid methyl ester | ↓ | D-Glucose |
| ↓ | 3-0-β-Galactopyranosyl-D-arabinose | ↓ | L-Asparagine |
| ↓ | pH 8 | ↓ | D-Glucosaminic acid |
| ↓ | pH 9.5 + cadaverine | ↓ | Tween 40 |
|  |  | ↓ | α-Keto-glutaric acid |
|  |  | ↓ | α-Keto-butyric acid |
|  |  | ↓ | α-Methyl-D-galactoside |
|  |  | ↓ | L-Glutamine |
|  |  | ↓ | m-Tartaric acid |
|  |  | ↓ | D-Fructose-6-phosphate |
|  |  | ↓ | Tween 80 |
|  |  | ↓ | α-Hydroxy butyric acid |
|  |  | ↓ | Glycyl-L-aspartic acid |
|  |  | ↓ | Citric acid |
|  |  | ↓ | D-Threonine |
|  |  | ↓ | Glycolic acid |
|  |  | ↓ | Glyoxylic Acid |
|  |  | ↓ | Glycyl-L-aspartic acid |
|  |  | ↓ | Citric acid |
|  |  | ↓ | D-Threonine |
|  |  | ↓ | Glycolic acid |
|  |  | ↓ | Glyoxylic acid |
|  |  | ↓ | Glycyl-L-glutamic acid |
|  |  | ↓ | Tricarballylic acid |
|  |  | ↓ | L-Serine |
|  |  | ↓ | Acetoacetic acid |
|  |  | ↓ | Monomethyl succinate |
|  |  | ↓ | D-Psicose |
|  |  | ↓ | L-Lyxose |
|  |  | ↓ | Glucuronamide |
|  |  | ↓ | D-Galacturonic acid |
|  |  | ↓ | D-Lactic acid methyl ester |
|  |  | ↓ | L-Pyroglutamic acid |
|  |  | ↓ | 6% NaCl + MOPS |
|  |  | ↓ | 6% NaCl +KCl |
|  |  | ↓ | 6% NaCl + N-acetyl L-glutamine |
|  |  | ↓ | 3% Sodium sulphate |
|  |  | ↓ | 4% Sodium sulphate |
|  |  | ↓ | 10% Ethylene glycol |
|  |  | ↓ | 15% Ethylene glycol |
|  |  | ↓ | 3% Sodium formate |
|  |  | ↓ | 2% Urea |
|  |  | ↓ | 3% Urea |
|  |  | ↓ | 5% Urea |
|  |  | ↓ | 6% Urea |
|  |  | ↓ | 7% Urea |
|  |  | ↓ | 8% Sodium lactate |
|  |  | ↓ | 100 mM Sodium phosphate pH 7 |
|  |  | ↓ | 200 mM Sodium benzoate pH 5.2 |
|  |  | ↓ | 10 mM Ammonium sulphate pH 8 |
|  |  | ↓ | 20 mM Ammonium sulphate pH 8 |
|  |  | ↓ | 50 mM Ammonium sulphate pH 8 |
|  |  | ↓ | 10 mM Sodium nitrate |
|  |  | ↓ | 20 mM Sodium nitrate |
|  |  | ↓ | 40 mM Sodium nitrate |
|  |  | ↓ | 60 mM Sodium nitrate |
|  |  | ↓ | 80 mM Sodium nitrate |
|  |  | ↓ | 100 mM Sodium nitrate |
|  |  | ↓ | pH 9.5 + L-Glutamine |
|  |  | ↓ | pH 9.5 + L-Tyrosine |
|  |  | ↓ | pH 9.5 + L-Norvaline |
|  |  | ↓ | X-α-D-Glucoside |
|  |  | ↓ | X-β-D-Glucoside |
|  |  | ↓ | X-α-D-Galactoside |

↑, increased growth of mutant relative to wild-type *C. jejuni* NCTC 11168;

↓, decreased growth of mutant relative to wild type *C. jejuni* NCTC 11168;

Cut-off for significant change in growth: ≥1.5-fold difference between wild-type and mutant for p <0.05; only statistically significant changes are included.
